# Opinionated Views on Genome-Assisted Inference and Prediction During a Pandemic

**DOI:** 10.3389/fpls.2021.717284

**Published:** 2021-08-06

**Authors:** Daniel Gianola

**Affiliations:** Department of Animal and Dairy Sciences, University of Wisconsin-Madison, Madison, WI, United States

**Keywords:** genomic selection, prediction, quantitative genomics, animal breeding, plant breeding, machine learning, bayesian methods, best linear unbiased prediction

Genome-assisted prediction of complex (e.g., quantitative) traits is an ingredient of “Genomic Selection,” a paradigm adopted successfully in animals and plants of agricultural importance. The approach has impacted the timing of selection decisions, and it has delivered improvements in the quality of predictions (“accuracy”) relative to what can be attained by the use of pedigrees and phenotypes. It has enhanced the rate of response to genetic selection and spectacularly so in dairy cattle, at least as suggested by genome-based estimates of genetic change. Researchers have spent much effort in developing and adapting prediction machines, and the author will focus on this matter, with mild excursions into tangential issues. The material is organized into nine sections and, since the author was to opine, it represents a set of personal views, rather than a review of literature, made retrospectively.

***1. Deconstruction of “genetic architecture”:*** Molecular genetics and biochemistry confirm that the theory of quantitative genetics provides just a linear (local) approximation to complexity with little (if any) mechanistic value. The intricate interactions and feedbacks inherent in biological systems cannot be captured by simple linear regressions, even if highly-dimensional regression models are fitted to the data. The effective dimension of a model cannot exceed the sample size. For example a model with 5 million parameters run with a sample size of 500 does not provide meaningful estimates of more than 500 distinct estimable functions of parameters: individual site effects are not likelihood-identified. The view that quantitative genomics can unravel the “genetic architecture” of complex traits by providing an inventory of allelic frequencies and allelic substitution effects, or by a decomposition of variance (typically complicated by strong linkage disequilibrium) is equivalent to stating that tons of bricks, steel, and glass can represent Zaha Hadid's new Beijing airport or Frank's Gehry's Guggenheim Museum at Bilbao. The author often refraines from using the buzz term “*genetic architecture”* and favores “*statistical architecture”* instead.

***2. Crumbs are not bread:*** The QTL paradigm [superseded by zillions of genome-wide association study (GWAS) in human genetics] has had a minor impact on agricultural practices (fertilization, management, etc.), with few exceptions. GWAS with single-marker regression is also insufficient because it accounts for little genetic variation (except for major effect variants at intermediate frequencies, which are “caught” by observation anyhow), apart from ignoring interactions as stated above. Although a more complex model may improve learning, the author has not seen reports where variable selection methods and members of the Bayesian alphabet capture signals much more effectively than a simple GWAS run with large samples, as in human genetics consortia. Shrinkage methods are typically “vector optimized” (with ridge regression notoriously so), and the borrowing of information facilitated by proper priors tends to make signals similar to each other. Bayesian variable selection (BVS) with spike-slab distributions may be more powerful, but signals from large-effect variants are strengthened at the expense of mitigating small effects. In BVS or LASSO, the “richer get richer and the poorer get poorer” whereas ridge regression is more “social democrat,” making effect-size estimates similar to each other. If a pizza for 500 persons is divided into 5 million unexpected guests, each will end up getting a crumb. The perception of the author is that advances in the resolution and causality of small-effect variants *via* GWAS and genome-enabled prediction have been marginal, at least in agriculture.

***3. Corroboration vs. induction:*** The main contributions of quantitative genetics (genomics) have been in description, prediction, and decision, e.g., selection choices, inbreeding management, and optimum contribution theory, as opposed to inference. In predictive approaches, genomic heritability or correlations take the role of “regularization knobs” (i.e., not viewed as parameters with existential meaning) for constructing prediction machines. The objective is to make statements about yet-to-be-observed phenotypes based on some training data. Predictions can be calibrated empirically, but inferences cannot. How can one say that an estimate of an entelechy, such as heritability is bad or good? Following Descartes: “*I cannot be observed, therefore, I do not exist*.” According to Encyclopedia Britannica, for Descartes to prove that heritability exists, one must assume it does. For prediction, “there is no need for that hypothesis” as often attributed to Laplace.

***4. Occam's razor resurrected:*** A less recognized but important ingredient of the study on genomic selection of Meuwissen et al. (2001), was the use of predictive cross-validation employed earlier in plant breeding but almost completely ignored in animal breeding. In the latter field, the ideas of Henderson (1963, 1973, 1984) encouraged work in developing more complex and bigger models, based on the (incorrect) perception that bigger was better. An example is multiple-trait longitudinal models for dairy cattle, producing cow-specific curves at the genetic and environmental levels for several lactations in hundreds of thousands of genetically related cows! Little attention had been devoted to evaluating whether or not a simple model would predict better than a bigger one. The use of cross-validation in genome-enabled prediction debunked the widespread perception. Big complex models make more assumptions and, with finite sample sizes, it is not uncommon that such models lack robustness, thus failing to deliver better predictions. During the 20th century, model choice received scant formal consideration in animal breeding, a notable exception being a study by Sorensen and Waagepetersen (2003). Genomic selection with cross-validation helped to refute older views. Simplicity can be effective and is often elegant.

***5. Prediction is inclusive:*** There is no universally best genome-based prediction machine for animal or plant breeding. The relative performance of the various methods depends mainly on the information content and structure of the training set, and on the extent to which a configuration of genotypes spanned in the training process will also appear in the testing set. These two aspects are difficult to evaluate *ex ante*. Often, the size of the training sample or functions thereof, e.g., Daetwyler et al. (2008), are used as a proxy for the “expected quality” of predictions. However, a sample may be huge and yet convey little information. The plant breeding group in Munich has worked (e.g., Auinger et al., 2021) in assessing genomic measures of information content, such as molecular diversity present in a training sample, and attempting to connect these metrics to predictive outcomes. For instance, a strong underlying structure may affect prediction adversely, even in large samples, so conceivably it could be modified to enhance the quality of outcomes. The larger the overlap between training and testing samples, the more relevant to a target population the statements made from training data will be. George Box and Norman Draper (my teacher in a regression course I took in 1972) taught: “*Never extrapolate beyond the experimental region”*. Suppose a prediction machine “sees” 50% *AABB* and 50% *aabb* individuals in the training process. However, the testing set has the configuration $\frac{1}{3}AABB+\frac{1}{3}AaBb+\frac{1}{3}aabb.$ Both sets have the same allelic frequencies, but the testing set contains a “novelty,” *AaBb*, so the prediction machine would be extrapolating. Genetic relatedness is a measure of such overlap, but the driving force is the degree of molecular similarity between individuals in the corresponding data partitions. Random replication of cross-validation may produce an estimate of an upper bound for predictive ability. Even when both training and testing sets are representative of a target population, the performance of prediction methods often depends on cryptic interplays between environment, trait and model complexity (effective number of parameters fitted vis-a-vis effective training sample size).

It is futile to have information-rich training samples but unrepresentative and ridiculously small testing sets, as large variation among outcomes of similar prediction exercises is to be expected. Small testing sets and failure to replicate cross-validation in some studies have produced results where models accommodating dominance and epistasis appear as delivering a somewhat better performance than additive prediction models. Such results may be “false positives” reflecting chance, rather than signal.

***6. And the Oscar goes to*…*:*** A simple method such as genomic best linear unbiased predictor (GBLUP) may tell something about the state of nature and perform adequately. An involved procedure, such as a deep neural network (DNN), may tell nothing and yet produce spectacular results, although it has failed miserably in some studies. Like all neural networks, a DNN is regarded as “universal approximator.” An ongoing meta-analysis of hundreds of studies made in INIA, Spain (disclaimer: I will be a coauthor) places reproducing kernel Hilbert spaces regression (RKHS); e.g., Wahba (2007) methods ahead of others, but only slightly. Work with animals and plants and with various field crops in CIMMYT (e.g., Costa-Neto et al., 2021) has shown the flexibility of kernel methods for capturing genome-environment interactions and environmental similarities. In Wisconsin, RKHS has been extended to the single-step BLUP setting, and the CIMMYT group is developing a multiple-trait Bayesian RKHS. Last, but not the least, RKHS is the mother of GBLUP, along the lines that Gibbs sampling is a child of the Metropolis-Hastings algorithm for Markov chain Monte Carlo sampling.

Animal breeding industries have embraced GBLUP, and there seems to be little scope for adoption of the Bayesian alphabet models (the membership of this club is converging to infinity) for routine use, but there can be exceptions. GBLUP is a “good thing” as pointed out in the early '90s, and we have known for a while that it is not only a special case of RKHS, but also a maximum penalized likelihood estimator, a linear neural network, and that it has a Bayesian interpretation. It has been extended to cross-sectional, multi-trait, longitudinal situations and has been “robustified.” Importantly, software developed mainly at the University of Georgia by Misztal allows crunching millions of predicted genomic breeding values. GBLUP with mild tweeks will probably remain the technology (term used deliberately) of choice for genetic evaluation of selection candidates. The science of genome-enabled prediction has arrived at a reasonable destination, but the voyage will continue, and new data will bring challenges.

***7. Help needed:*** Despite an abundance of chips enabling large-scale genotyping, training samples are seldom drawn at random, thus unrepresentative. This situation constitutes a selection process that is often not considered in predictive models. Animal breeders have been widely influenced by the “selection bias” study of Henderson (1975), based on questionable assumptions, as pointed out first by Robin Thompson (1979). *Ad-hoc* approaches and arguments have been used for justifying some forms of analysis or modeling, such as the notion of treating a large number of contemporary groups as fixed, leading to inefficient estimation (James-Stein “inadmissibility” argument; Judge et al., 1985). The arguments were based on an obscure notion of bias removal advocated by Henderson. Such views have carried into genome-enabled prediction in animal breeding. The problem of employing selected samples for inference and prediction stands and should be studied with more rigor, e.g., *via* missing data theory.

***8. Bias against bias:*** The notion of statistical “bias” continues to be misunderstood. GBLUP is believed (just consider its name) to be an unbiased predictor, but it is identical to ridge regression in some settings. However, ridge regression is a biased estimator. Is this a Dr. Jekyll Mr. Hide issue or some statistical bipolarity? The answer is that prediction and estimation unbiasedness have different definitions! Say you own a plant or a bull called “Charlie,” a fixed entity with identity (e.g., if Charlie is *AA*, it has a specific breeding value that possibly differs from that of *Aa* or *aa*). You are not interested in learning the average of a (very) large sample of potential Charlies; rather, you seek the breeding value of the Charlie you have. If ridge regression is used to estimate the breeding value of Charlie, Dr. Jekyll says there is estimation bias, but Mr. Hide states that there would be none. The latter is wrong (in the bias sense) with respect to Charlie, but not with respect to an average of potential Charlies, some of which will be *AA*, some *Aa*, and some *aa*. All good members of the Bayesian alphabet including GBLUP, with its appealing Bayesian interpretation (Gianola and Fernando, 1986), and practically all machine learning methods (e.g., random forests) provide biased predictions that, on average, will be better than unbiased machines. A potential therapy for unbiasedness-obsession is “debiasing” (Breiman, 2001). However, predictions would be probably worse because, in addition to the extant uncertainty of prediction sets, there would be an extra error resulting from a deteriorated bias-variance trade-off. In the end, the debiased genome-enabled predictions may be much worse than prior to bias removal.

***9. Use a GPS to map the road ahead:*** Defining pertinent breeding objectives (the classical Smith-Hazel problem) continues being crucial in practice, but it has become academically non glamorous at these times of massive genotyping, epigenotyping, proteomics, metabolomics, and (fine) phenotyping. It is important not to lose perspective as, otherwise, breeders will get inebriated with a cocktail “on apps.” Another issue of (some) concern is that the current emphasis on “big data,” “massive computing,” and “visualization” may diminish basic science education, as it appears that current thesis students start crunching numbers before they know genomics, or the meaning of a probability distribution, or attain an elementary knowledge of randomization or causality. Foundational theory and concepts should continue being taught. Otherwise, the field may drown in a technology-induced maelstrom, and critical or even visionary perspectives may end up playing a role that becomes secondary to that of a beautiful visualization or, even worse, to a machine.

W. G. Hill (“Bill”) noted in a 2010 study discussing from Lush to Genomics: “Opinions we can debate.” I look forward to that conversation (Hill, 2010).

## Author Contributions

DG prepared the first draft of the opinion, provided critical comments, typed the definitive version, and checked the spelling. The author contributed to the article and approved the submitted version.

## Conflict of Interest

The author declares that the research was conducted in the absence of any commercial or financial relationships that could be construed as a potential conflict of interest. The handling editor declared a past collaboration with the author DG.

## Publisher's Note

All claims expressed in this article are solely those of the authors and do not necessarily represent those of their affiliated organizations, or those of the publisher, the editors and the reviewers. Any product that may be evaluated in this article, or claim that may be made by its manufacturer, is not guaranteed or endorsed by the publisher.
